# Cathepsin B & L Are Not Required for Ebola Virus Replication

**DOI:** 10.1371/journal.pntd.0001923

**Published:** 2012-12-06

**Authors:** Andrea Marzi, Thomas Reinheckel, Heinz Feldmann

**Affiliations:** 1 Laboratory of Virology, Division of Intramural Research, National Institute of Allergy and Infectious Diseases, National Institutes of Health, Hamilton, Montana, United States of America; 2 Special Pathogens Program, National Microbiology Laboratory, Public Health Agency of Canada, Winnipeg, Manitoba, Canada; 3 Institute of Molecular Medicine and Cell Research, Albert-Ludwigs-University, Freiburg, Germany; 4 BIOSS Centre for Biological Signaling Studies, Albert-Ludwigs-University, Freiburg, Germany; 5 Department of Medical Microbiology, University of Manitoba, Winnipeg, Manitoba, Canada; University of Texas Medical Branch, United States of America

## Abstract

Ebola virus (EBOV), family *Filoviridae*, emerged in 1976 on the African continent. Since then it caused several outbreaks of viral hemorrhagic fever in humans with case fatality rates up to 90% and remains a serious Public Health concern and biothreat pathogen. The most pathogenic and best-studied species is *Zaire ebolavirus* (ZEBOV). EBOV encodes one viral surface glycoprotein (GP), which is essential for replication, a determinant of pathogenicity and an important immunogen. GP mediates viral entry through interaction with cellular surface molecules, which results in the uptake of virus particles via macropinocytosis. Later in this pathway endosomal acidification activates the cysteine proteases Cathepsin B and L (CatB, CatL), which have been shown to cleave ZEBOV-GP leading to subsequent exposure of the putative receptor-binding and fusion domain and productive infection. We studied the effect of CatB and CatL on *in vitro* and *in vivo* replication of EBOV. Similar to previous findings, our results show an effect of CatB, but not CatL, on ZEBOV entry into cultured cells. Interestingly, cell entry by other EBOV species (*Bundibugyo*, *Côte d'Ivoire*, *Reston* and *Sudan ebolavirus*) was independent of CatB or CatL as was EBOV replication in general. To investigate whether CatB and CatL have a role *in vivo* during infection, we utilized the mouse model for ZEBOV. Wild-type (control), *catB^−/−^* and *catL^−/−^* mice were equally susceptible to lethal challenge with mouse-adapted ZEBOV with no difference in virus replication and time to death. In conclusion, our results show that CatB and CatL activity is not required for EBOV replication. Furthermore, EBOV glycoprotein cleavage seems to be mediated by an array of proteases making targeted therapeutic approaches difficult.

## Author Summary

It is currently believed that Ebola virus (EBOV) enters cells via macropinocytosis following which, the cysteine proteases cathepsin B and L (CatB, CatL) cleave the viral glycoprotein (GP) allowing exposure of its core receptor-binding and fusion domain thus facilitating subsequent infection. We studied the effect of CatB and CatL on *in vitro* and *in vivo* EBOV replication. Our results demonstrate a reduction of *Zaire ebolavirus* (ZEBOV) entry upon selective inhibition of CatB, but not CatL in cell culture. Interestingly, all other EBOV species enter the cells efficiently when CatB and/or CatL activity is blocked. Moreover, when wild-type (control), *catB^−/−^* and *catL^−/−^* mice were infected with a lethal dose of mouse-adapted ZEBOV, all animals were equally susceptible to lethal challenge with no difference in virus replication and time to death. Therefore, we conclude that EBOV replication is dispensable of CatB and CatL, and proteolytic processing of GP can also be mediated by other endosomal proteases.

## Introduction

Members of the family *Filoviridae*, Ebola virus (EBOV) and Marburg virus (MARV), are the causative agents of viral hemorrhagic fever in central Africa and a major public health threat in their endemic areas. Worldwide concern relates to the importation of infected individuals and the potential use of filoviruses as biothreat pathogens [1]. All current MARV strains belong to the *Lake Victoria marburgvirus* species, while Ebola virus (EBOV) strains are attributed to five different species: *Zaire ebolavirus* (ZEBOV), *Sudan ebolavirus* (SEBOV), *Côte d'Ivoire ebolavirus* (CIEBOV), *Reston ebolavirus* (REBOV) and *Bundibugyo ebolavirus* (BEBOV) [1],[2]. The species vary in their pathogenicity for humans with ZEBOV being most pathogenic (up to 90% case fatality rate), followed by SEBOV and BEBOV with about 50% and >25% case fatality rates, respectively. CIEBOV and REBOV cause lethal infections in nonhuman primates, but have not yet been associated with fatal human cases [1], [2]. Although EBOV (mainly ZEBOV) and MARV have been extensively studied *in vitro* and *in vivo*, today there is neither a licensed vaccine nor treatment available.

EBOV entry into target cells is still not fully understood and remains a focus of ongoing research. While a number of attachment factors seem to facilitate EBOV entry [3], so far no specific cell surface receptor molecule has been identified. Recently, it was shown that the cholesterol transporter Niemann-Pick C1 (NPC1) is required for ZEBOV infection [4], [5]. NPC1 is a ubiquitously expressed endosomal membrane protein involved in the fusion and fission of endosomes and lysosomes [6], and its deficiency has been shown to impact on HIV-1 particle release [7]. Its presence in the endosome fits the current EBOV entry model based on macropinocytosis, a cellular pathway proposed to be the main uptake mechanism of EBOV particles into cells [8]–[10]. Previously, Chandran *et al.* identified the cysteine proteases cathepsin B (CatB) and cathepsin L (CatL), which are also present in endosomes, as important factors for ZEBOV entry [11]. According to the current model, cleavage of the ZEBOV glycoprotein (GP) by CatB is necessary for exposure of the core receptor-binding domain and fusion machinery, otherwise buried in the GP structure, to initiate fusion of the viral and the endosomal membrane [12]–[14]. Subsequently, the viral genome along with the replication complex is released into the cytoplasm where replication and virus progeny production occur.

CatB and CatL are members of a family of 11 human cysteine proteases. Both proteases are highly abundant, broadly expressed and exhibit nonspecific proteolytic activity within lysosomes [15], [16]. CatB has been associated with TNF-α induced liver damage and seems to play a critical role for the development of pancreatitis [17],[18]. Despite these facts, *catB^−/−^* mice are phenotypically similar to wild-type control mice and fully immunocompetent [18]. CatL is important for epidermal homeostasis and the regulation of the hair cycle and as such *catL^−/−^* mice are hairless [19]. Furthermore, CatL is involved in MHC II-mediated antigen presentation in epithelial cells of the thymus [20]. Consequently, *catL^−/−^* mice have reduced numbers of CD4+ T helper cells, which are however fully functional. A double knockout mouse lacking CatB and CatL has been generated but is not viable long enough for experimental use [21], [22]. To determine the importance of cathepsins in viral infections, various inhibitors of endosomal acidification, cathepsin or specifically CatB and CatL activity have been used *in vitro*. In these studies the role of cathepsins have been demonstrated to be important for the entry of reovirus, SARS-CoV, henipaviruses and ZEBOV [11], [14], [23]–[27].

Here we show that while CatB mediates ZEBOV entry *in vitro*, it is not important for entry and replication of any other EBOV species. We further show that *in vivo* replication of mouse-adapted ZEBOV (MA-ZEBOV) is independent of CatB and CatL, indicating that both of these cathepsins are not required for ZEBOV replication *in vivo*.

## Methods

### Animal ethics statement

Animals were handled in the Biosafety Level 4 (BSL4) containment space of the Integrated Research Facility (IRF) at the Rocky Mountain Laboratories (RML), Division of Intramural Research (DIR), National Institute of Allergy and Infectious Diseases (NIAID), National Institutes of Health (NIH). Research was conducted in compliance with the guidelines of the NIAID/RML Institutional Animal Care and Use Committee (IACUC). The facility, where this research was conducted, is fully accredited by the Association for the Assessment and Accreditation of Laboratory Animal Care International (AAALAC) with approved Office of Laboratory Animal Welfare (OLAW) assurance (#A4149-01). Research was conducted under a protocol approved by the IACUC. All procedures were conducted by trained personnel under veterinary supervision, and all invasive clinical procedures were performed while animals were anesthetized. Endpoint criteria, as specified by the IACUC approved scoring parameters, were used to determine when animals should be humanely euthanized.

### Cell culture and virus propagation

Vero E6 cells and Mouse Embryonic Fibroblast (MEF) cell lines lacking cathepsin B, cathepsin L or both cathepsins were cultured in Dulbecco's Modified Eagle Medium (DMEM) (Sigma, St. Louis, MO) supplemented with 10% FBS, penicillin/streptomycin and L-glutamine in a 37°C incubator, 5% CO_2_. VSV (serotype Indiana), SARS (strain Tor 2), BEBOV [2], CIEBOV (strain Tai Forest), REBOV (strain Pennsylvania), SEBOV (strain Boniface), ZEBOV (strain Mayinga) and the furin knockout and mouse-adapted variants of ZEBOV (ZEBOV-Fko and MA-ZEBOV, respectively) [28]–[30] were propagated in Vero E6 cells. The supernatants were cleared of cell debris by centrifugation at 1,500×g for 10 min, aliquoted and stored in liquid nitrogen. Viral titers were determined conducting conventional plaque assay or immunoplaque assay. All infectious work with EBOV was performed in the Biosafety Level 4 (BSL4) laboratories at the National Microbiology Laboratory (NML) of the Public Health Agency of Canada (PHAC) or the IRF, RML, DIR, NIAID, NIH.

### Foci reduction experiments

The endosomal acidification inhibitor Bafilomycin A1 (BafA1) and the inhibitor CA074 (specific for CatB) were obtained from Sigma (St. Louis, MO); CatL-inhibitor V was purchased from Calbiochem (via EMD Chemicals Inc., Gibbstown, NJ). All inhibitors were dissolved in DMSO and stored at −20°C. Dilutions were prepared in plain DMEM (no supplements) prior to every experiment. For foci reduction experiments Vero E6 cells were seeded in 48-well plates the day before infection. Media was removed and cells were incubated for one hour with 50 µl inhibitor in the following concentrations as described previously [11]: BafA1 200, 100, 50, 20 nM; CA074 (CatB) 200, 100, 50, 20 µM; CatL-inhibitor V 20, 10, 5, 2 µM. Then 50 µl plain DMEM with 100 focus forming units (ffu) virus were added and kept for one hour at 37°C. After three washes with plain DMEM a 1∶1 mixture of 2.4% carboxymethyl cellulose (CMC) and 2× Minimal Essential Medium (MEM) (Life Technologies, Carlsbad, CA) supplemented with 4% FBS, penicillin/streptomycin and L-glutamine was added containing the appropriate concentration of inhibitor (BafA1 100, 50, 25, 10 nM; CA074 (CatB) 100, 50, 25, 10 µM; CatL-inhibitor V 10, 5, 2.5, 1 µM). SARS infected cells were fixed and stained with crystal violet and plaques were counted three days after infection. Four days after infection EBOV inoculated cells were fixed with 10% neutral buffered formalin and removed from BSL4 following standard operating procedures. Subsequently, the cells were permeabilized and foci were stained with a rabbit anti-VP40 antibody (kindly provided by Y. Kawaoka, University of Wisconsin, Madison, WI) or a rabbit serum directed against REBOV-NP (kindly provided by A. Takada, Hokkaido University, Sapporo, Japan) followed by a FITC-labeled secondary antibody (Sigma, ST. Louis, MO). Foci were counted using a fluorescent microscope (Carl Zeiss Microimaging LLC, Thornwood, NY).

### Viral growth kinetics

Vero E6 cells were seeded in a 24-well plate the day before the experiment. Pretreatment occurred with 200 µl of 100 nM BafA1, 100 µM CA074, 10 µM CatL-inhibitor V or no inhibitor for 1 hour. Thereafter, 200 µl ZEBOVwt (MOI = 1) were added and cells were incubated for another hour. Following three washes with plain DMEM, cells in each well were covered with 1 ml DMEM (supplemented with 2% FBS) containing 50 nM BafA1, 50 µM CA074, 5 µM CatL-inhibitor V or no inhibitor. MEF cell lines were seeded in a 24-well plate the day before the experiment. For infection, 200 µl ZEBOVwt or MA-ZEBOV (MOI = 1) were added and incubated for one hour. Following three washes with plain DMEM, cells in each well were covered with 1 ml DMEM (supplemented with 2% FBS). At time points 0, 12, 24, 48, 72 and 96 hours post infection 200 µl supernatant were collected from all infected cells, and 200 µl DMEM with 2% FBS containing the appropriate concentration of inhibitor were added back into each well. Samples were stored at −80°C before titration on Vero E6 cells.

### Mouse infections

Groups of C57BL/6 *catB^−/−^*, C57BL/6 *catL^−/−^* or C57BL/6 (control) mice were infected intraperitoneally (i.p.) with 10 ffu MA-ZEBOV (1,000 LD_50_) or 1×10^5^ pfu VSVwt (serotype Indiana) and monitored daily for weight loss and signs of disease. On day 3 and 7 post infection 3 mice of each group were euthanized and blood, liver and spleen samples were collected and stored at −80°C for virus titration. Surviving animals were euthanized at day 28 (study endpoint) and final serum was collected to determine antibody titers.

### Virus load determination

Vero E6 cells were seeded in 96-well plates the day before titration. Liver and spleen samples were thawed, weighed, homogenized in 10-fold weight/volume of plain DMEM and serial 10-fold dilutions were prepared. Blood samples and supernatant collected from infected cells to determine viral growth for ZEBOVwt and MA-ZEBOV were thawed and serial 10-fold dilutions were prepared. Media was removed from cells and triplicates were inoculated with each dilution. After one hour DMEM supplemented with 2% FBS, penicillin/streptomycin and L-glutamine was added and incubated at 37°C. Cells were monitored for cytopathic effect (CPE) and 50% tissue culture infectious dose (TCID_50_) was calculated for each sample employing the Reed and Muench method [31].

### Humoral immune responses in mice

For the detection of ZEBOV-GP specific antibodies in mouse sera, soluble ZEBOV-GP antigen (ZEBOV-GPΔTM) was produced and used in an enzyme-linked immunosorbent assay (ELISA) as described before [32], [33]. For the detection of VSV-specific antibodies VSV was propagated in Vero E6 cells. VSV particles were harvested after 48 hrs, purified by centrifugation through a 20% sucrose cushion and treated with 0.05% Triton-X100 in PBS prior to storage at −80°C. The ELISA used VSV antigen in a 1∶100 dilution. Sera from mice infected with MA-ZEBOV were inactivated by γ-irradiation as per standard operating protocol.

### Statistical analyses

One-way ANOVA was performed using Prism 5 (Graph Pad Software Inc.).

## Results

### ZEBOV entry *in vitro* is CatB-mediated

**Figure 1:**
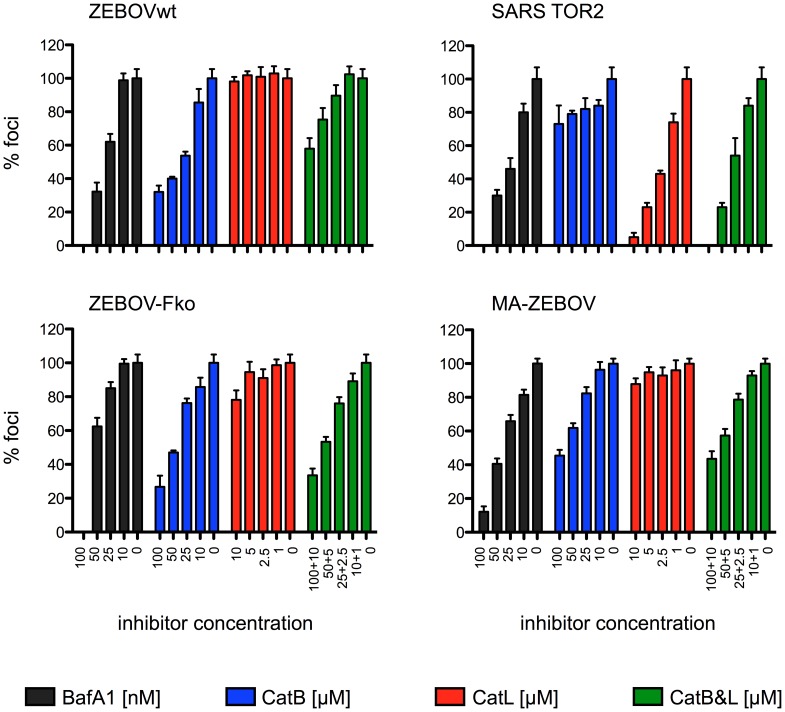
*Zaire ebolavirus* entry is CatB mediated. Vero E6 cells were treated prior to infection for one hour with inhibitors directed against the indicated cathepsin(s) or endosomal acidification (BafA1). Following infection the cells were washed and a carboxymethyl cellulose overlay was added containing inhibitor (BafA1 100, 50, 25, 10 nM; CA-074 (CatB) 100, 50, 25, 10 µM; CatL inhibitor V 10, 5, 2.5, 1 µM). SARS-CoV infected cells were fixed on day 3, stained with crystal violet and plaques were counted. ZEBOV infected cells were fixed on day 4, foci were stained with an antibody against ZEBOV-VP40 and counted. The number of foci and plaques without inhibitor was set as 100%. A representative experiment performed in triplicates is shown. Error bars indicate the standard error of the mean. ZEBOVwt = *Zaire ebolavirus*, strain Mayinga; ZEBOV-Fko = *Zaire ebolavirus* furin cleavage site knockout mutant; MA-ZEBOV = mouse-adapted *Zaire ebolavirus*.

The observation that entry of ZEBOV into Vero E6 cells is CatB- and CatL-dependent was initially described in 2005 by Chandran and colleagues [11]. For further analysis of ZEBOV uptake into Vero E6 cells we performed a different *in vitro* assay based on foci reduction using the same inhibitors. Cells were seeded and treated with various lysosome and cathepsin inhibitors for one hour, infected with ZEBOV wild-type (wt) for one hour, covered with carboxymethyl cellulose containing inhibitors and fixed after incubation for 4 days. The number of foci was determined by immunostaining. Only the inhibition of CatB resulted in significantly reduced virus entry, whereas CatL proteolytic activity did not appear to be obligatory for ZEBOV uptake into Vero E6 cells, although CatL is highly expressed in these cells [27]. SARS-CoV was used as a CatL-dependent control virus [26] to verify the activity of the CatL inhibitor (Fig. 1). In addition to ZEBOVwt, we also tested a furin cleavage site knockout ZEBOV (ZEBOV-Fko) [28], [29] and the MA-ZEBOV in this assay to determine whether furin cleavage of the glycoprotein or mutations caused by the adaptation process of the virus to mice would influence the requirement for cathepsin cleavage. Both viruses entered Vero E6 cells in a CatB-dependent manner similar to ZEBOVwt and were not affected by the presence of the CatL inhibitor (Fig. 1). These results show that the uptake of all tested viruses occurred by utilizing the cellular endocytotis machinery as indicated by the inhibitory effect of Bafilomycin A1 (BafA1), an endosomal acidification inhibitor. Furthermore, proteolytic processing of the ZEBOV-GP by furin is not a prerequisite for cathepsin cleavage in the virus maturation or entry process. In addition, mutations throughout the viral genome acquired during the adaptation process of ZEBOV to the mouse have no influence on ZEBOV uptake.

### The role of CatB in cellular uptake differs among Ebola virus species

**Figure 2:**
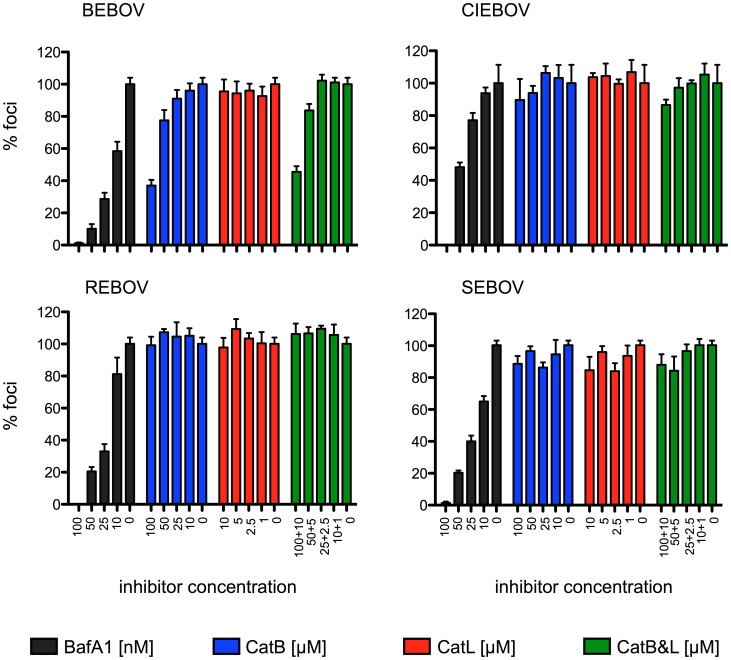
Entry of other Ebola viruses is CatB-independent. Vero E6 cells were treated prior to infection for one hour with inhibitors directed against the indicated cathepsin(s) or endosomal acidification (BafA1). Following infection the cells were washed and a carboxymethyl cellulose overlay containing inhibitor (BafA1 100, 50, 25, 10 nM; CA-074 (CatB) 100, 50, 25, 10 µM; CatL inhibitor V 10, 5, 2.5, 1 µM) was added. After fixation on day 4, foci were stained with antibodies against VP40 (BEBOV = *Bundibugyo ebolavirus*, CIEBOV = *Côte d'Ivoire ebolavirus*, SEBOV = *Sudan ebolavirus*, strain Boniface) or NP (REBOV = *Reston ebolavirus*, strain Pennsylvania) and counted. The number of foci without inhibitor was set as 100%. A representative experiment performed in triplicates is shown. Error bars indicate the standard error of the mean.

Our data on ZEBOV only partially support previously published observations [11], but confirm more recent studies claiming that CatB, rather than CatL, plays a role in ZEBOV entry [34], [35]. Therefore, we studied the role of cathepsin cleavage on virus entry for BEBOV, CIEBOV, REBOV and SEBOV using a representative strain for each EBOV species. Foci reduction assays were performed and evaluated as described above. Infection of all viruses was reduced in the presence of BafA1 (Fig. 2), confirming that endosomal acidification plays a critical role during the entry process of all these EBOVs. Interestingly, only the maximum concentration of the CatB inhibitor had an effect on BEBOV uptake into Vero E6 cells, whereas CIEBOV, REBOV and SEBOV produced foci independent of the presence of the inhibitor (Fig. 2). The entry of none of the EBOVs was affected by the CatL inhibitor. This data suggests that CatB is not equally important for the entry of other EBOVs and seems to have a selective effect on ZEBOV and a limited effect on BEBOV entry.

### ZEBOV replication is largely independent on cathepsin B cleavage

**Figure 3:**
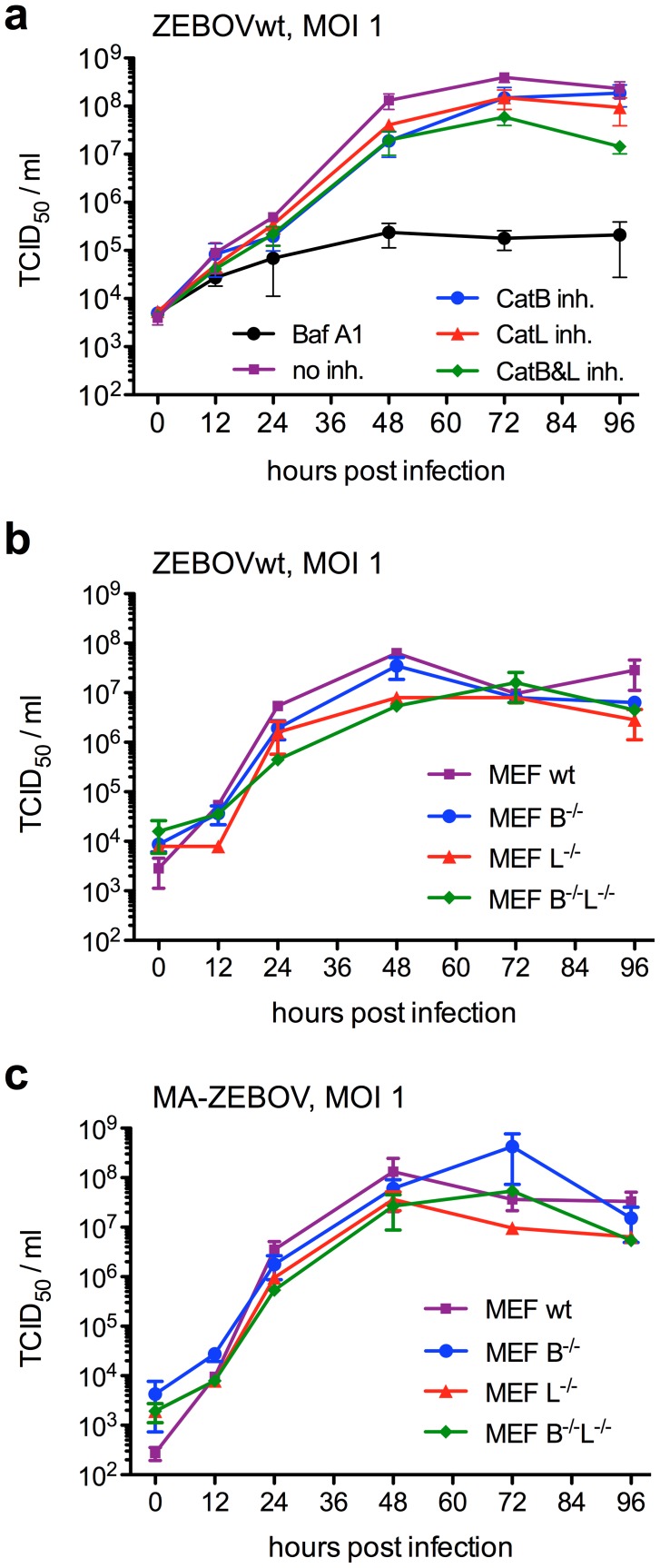
*Zaire ebolavirus* growth is CatB and CatL-independent. (**A**) Vero E6 cells were seeded the night before infection in a 24-well-plate. One hour prior to infection cells were incubated with inhibitors directed against the indicated cathepsin(s) or endosomal acidification (Baf A1). ZEBOVwt was added at an MOI of 1 and incubated for one hour at 37°C. After three washes the cells were covered with 1 ml medium containing 50 nM BafA1, 50 µM CA-074, 5 µM CatL inhibitor V or no inhibitor and incubated for 4 days. (B) and (C) MEF cell lines were seeded the night before infection in a 24-well-plate. Cells were infected for 1 hour with 0.2 ml ZEBOVwt (B) or MA-ZEBOV (C) at a MOI of 1. After three washes the cells were covered with 1 ml medium and incubated for 4 days. For all experiments, samples were collected at 0, 12, 24, 48, 72 and 96 hours post infection and infectious titers were determined. A representative experiment performed in triplicates is shown. Error bars indicate the standard error of the mean.

After confirmation that CatB has a role in ZEBOV entry we analyzed virus replication in the presence of cathepsin inhibitors. Vero E6 cells were treated with inhibitors (BafA1 100 nM; CatB 100 µM; CatL 10 µM) for one hour prior to ZEBOVwt infection (MOI = 1). After unbound virus was washed away the cells were covered with medium containing inhibitor (BafA1 50 nM; CatB 50 µM; CatL 5 µM), samples were taken at the indicated time points and stored at −80°C for virus titration. Virus titers were determined on Vero E6 cells using a 50% tissue culture infectious dose (TCID_50_) assay and calculated using the Reed and Muench formula (Fig. 3A) [31]. The results demonstrate that ZEBOV replicates to similar titers in the presence of CatB or CatL inhibitor (difference less than 1 log); the presence of both inhibitors reduced ZEBOV replication by about 1 log. This indicates that the CatB inhibitory effect on entry is likely compensated for by other cellular proteases. However, BafA1 reduces virus growth by more than 2 logs, showing that endosomal acidification is important for efficient ZEBOV entry and replication (Fig. 1, 3A). In order to further confirm the data we performed ZEBOVwt and MA-ZEBOV infections in MEF cell lines deficient in the expression of CatB, CatL or both cathepsins. The cells were infected for one hour with ZEBOVwt or MA-ZEBOV (MOI = 1) and samples were taken at the indicated time points. Virus titers were determined on Vero E6 cells using a TCID_50_ assay and calculated using the Reed and Muench formula (Fig. 3B,C) [31]. ZEBOVwt and MA-ZEBOV replicated similarly well in the absence of CatB or CatL or both proteases. Although small differences in titer were observed at certain time points, one-way ANOVA did not find statistically significant *p* values (Fig. 3).

### *CatB^−/−^* and *catL^−/−^* mice succumb to ZEBOV infection

**Figure 4:**
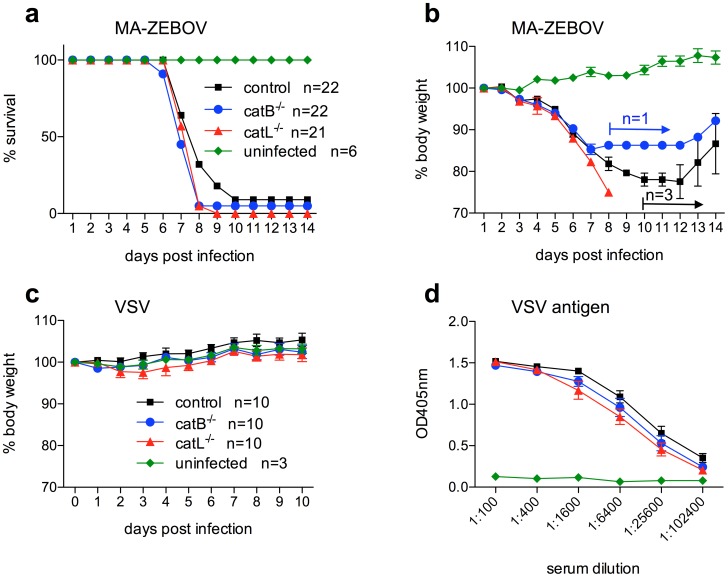
*CatB^−/−^* and *catL^−/−^* mice succumb to Ebola virus but not to VSV infection. Groups of mice were i.p. infected with 10 ffu MA-ZEBOV (1,000 LD_50_) or 1×10^5^ pfu VSV (serotype Indiana) and monitored daily for weight loss and other signs of illness. Survival (**A**) and weight curves (**B**) for MA-ZEBOV infection are shown. Body weights of VSV-infected mice are shown in (**C**). VSV antibodies were detected using ELISA to confirm infection (**D**).

**Figure 5:**
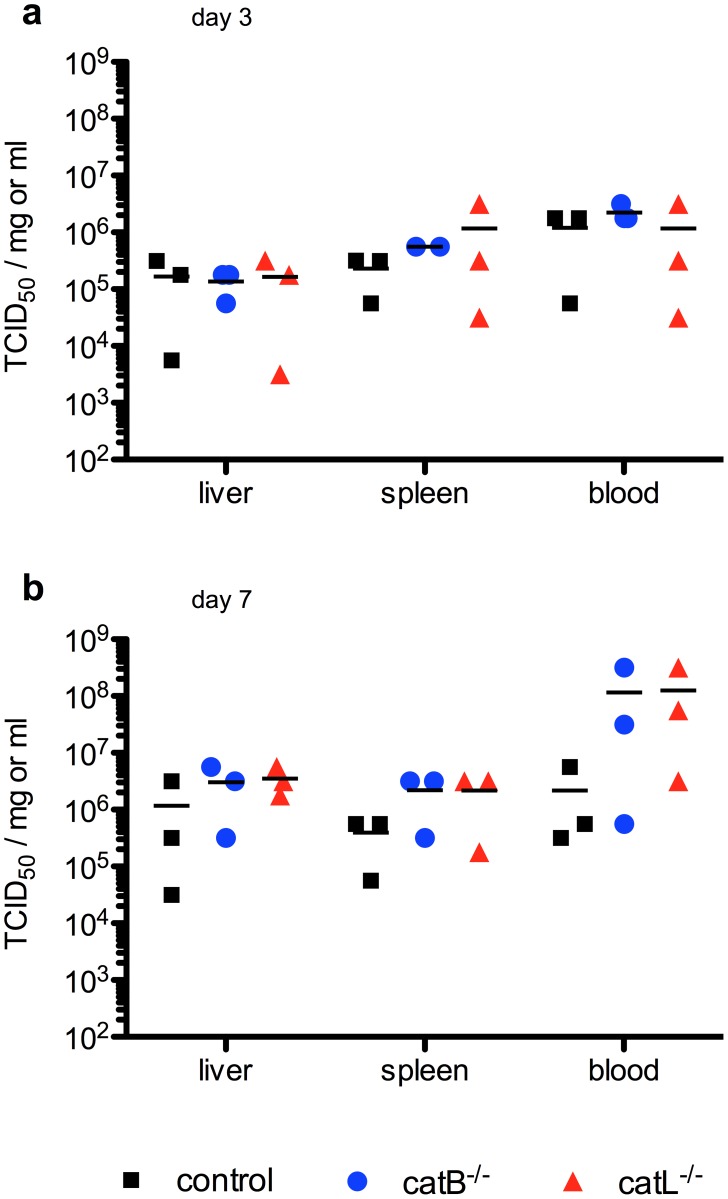
*Zaire ebolavirus* replicates to similar titers in knockout and control mice. *CatB^−/−^*, *catL^−/−^* or control mice (n = 3) were i.p. infected with 1,000 LD_50_ of MA-ZEBOV and euthanized at the indicated time point. Liver, spleen and blood samples were taken on day 3 (**A**) and day 7 (**B**) and viral titers were determined. A single 50% tissue culture infectious dose (TCID_50_) value is depicted for each mouse. Bars indicate the mean value.

Finally, the *in vitro* data were verified *in vivo* using the well-established lethal mouse model for ZEBOV [36]. The *catB^−/−^* and *catL^−/−^* mice are well characterized [37] and therefore ideal to determine the importance of CatB and CatL for ZEBOV replication *in vivo*. MEF cell lines obtained from these mice have been used here and in previous studies to demonstrate that mouse cathepsins are functionally similar to human cathepsins and important for ZEBOV-GP-mediated entry [11], [38]. Groups of *catB^−/−^*, *catL^−/−^* and control mice were infected with 1,000 LD_50_ of MA-ZEBOV and monitored daily for signs of disease including weight loss (Fig. 4A,B). On day 3 and 7 post-infection 3 mice in each group were euthanized and blood, liver and spleen samples were taken to determine the viral load (Fig. 5). All animals in the *catL^−/−^* group succumbed to MA-ZEBOV infection between days 7 and 9, similar to most control mice. All but one of the 22 *catB^−/−^* mice challenged with MA-ZEBOV succumbed to infection; the one surviving mouse showed signs of disease and recovered (Fig. 4A,B). As previously observed, the infection of control mice is not always uniformly lethal [33]. Here 3 out of 22 control mice developed disease but survived the challenge (Fig. 4A,B). There were no significant differences between viral titers in liver, spleen and blood samples taken on day 3 post MA-ZEBOV infection from the three different mouse strains (Fig. 5A). At day 7 post Ma-ZEBOV infection liver and spleen titers were similar among all three mouse strains but higher in the blood of *catB^−/−^* and *catL^−/−^* mice compared with wt mice (Fig. 5B). This indicates that disease progression and outcome were similar in all the animals (Fig. 4, 5). In order to exclude the occurrence of mutations in the glycoprotein (GP) gene of MA-ZEBOV during *in vivo* replication, we determined the full GP sequence and found no mutations (data not shown).

To exclude that *catB^−/−^* and *catL^−/−^* mice are in general more susceptible to viral infections, groups of mice were infected with recombinant vesicular stomatitis virus (VSV), strain Indiana, and monitored daily for weight loss and signs of illness (Fig. 4C). VSV did not cause disease in any of the mice demonstrating that neither the *catB^−/−^* nor the *catL^−/−^* mice do possess an increased susceptibility to virus infection. In blood, liver or spleen samples taken on day 3 and 7 post VSV infection no viral RNA was detected (data not shown), indicating that *catB^−/−^* and *catL^−/−^* as well as control mice were able to efficiently clear the virus. ELISA performed with serum samples of these mice showed that all animals were infected as indicated by the detection of VSV-specific antibodies (Fig. 4D). This data demonstrates that there is no obvious difference between *catB^−/−^*, *catL^−/−^* and control mice in susceptibility to viral infections and the development of immune responses.

## Discussion

The present study demonstrates that CatB, but not CatL, mediates ZEBOV uptake into Vero E6 cells. This observation is only partially in line with the initial *in vitro* studies demonstrating both CatB and CatL dependent uptake of ZEBOV [11], [14] and supports more recent studies showing that only CatB mediates ZEBOV entry into target cells [34], [35]. In addition, we could demonstrate that post-translational furin cleavage of ZEBOV-GP into the fragments GP_1_ and GP_2_ is not a prerequisite for cathepsin processing. This result does not come as a big surprise considering earlier studies reporting that ZEBOV replication and pathogenicity were independent of furin cleavage [28], [29].

Interestingly, all other EBOV species tested in our study seem to enter Vero E6 cells in a CatB- and CatL-independent manner suggesting that other endosomal proteases might functionally replace CatB in virus entry. This finding is in disagreement with recently published data showing that cell entry of VSV- and HIV-1-based pseudotype particles expressing different EBOV-GPs is CatB-dependent [38], [39]. Interestingly, one of these studies also showed that cell entry of infectious SEBOV (strain Gulu) was CatB- and CatL-independent as we could demonstrate here for a different SEBOV strain (strain Boniface) [38]. The discrepancies among our data and some of the previously published reports might be explained by the differences in size and shape of particles as well as the mechanism of particle uptake. HIV-1 particles are largely spherical and the mechanism of uptake is receptor mediated through the interaction of its surface glycoprotein gp160 with CD4 and CCR5 or CXCR4 [40]. VSV particles are short and bullet-shaped and cell uptake occurs via the endocytic pathway [41]. In the infectious ZEBOV context the interactions of GP with VP24 and VP40 (missing in pseudotype particles) may further influence the cellular uptake mechanism [14], altogether suggesting that HIV-1- and VSV-based pseudotype particle entry could be different from those of filovirus particles, which are extremely long and filamentous in shape and mainly utilize macropinocytosis for particle uptake [8]–[10]. In addition, the CIEBOV-GP used to produce VSV-based pseudotype particles in one study lacked the mucin-like domain, which could have had impact on the GP structure and thus might have affected cleavage and entry [38]. In our view this highlights the need for confirmation of data obtained from pseudotype particle systems by live EBOV infections or at least by the use of EBOV-like particles. Finally, cell type and origin may also influence CatB- and CatL-mediated cleavage as studies were performed in different cell lines.

For SARS-CoV entry, which is reported to be highly CatL-dependent, it has been shown that expression of the cellular transmembrane protease serine 2 (TMPRSS2) can overcome the block in SARS-CoV infection and replication caused by CatL inhibitors [42]–[45]. Moreover, signaling of toll-like receptor 9 (TLR9) has initially been associated with CatB, CatL and CatK activities [46], [47]. However, studies using bone marrow derived macrophages and dendritic cells derived from cathepsin knockout mice did not identify a single cathepsin as an essential factor for TLR9 signaling [48], [49] and rather point towards a role of other endolysosomal proteases, such as asparagine endopeptidase (AEP) [50] for activation. Thus, it seems reasonable to speculate that in the absence of CatB and CatL, such as in corresponding knockout mice, other endosomal proteases will mediate EBOV-GP cleavage enabling cathepsin-independent EBOV entry into target cells.

Previous reports have shown that EBOV-GPs were also processed by cathepsins in MEFs indicating that mouse CatB and CatL are functionally active [11], [38]. Here we have demonstrated that, similarly to ZEBOVwt, cell entry by MA-ZEBOV into Vero E6 cells was CatB-dependent but CatL-independent (Fig. 1). Furthermore, both EBOVs replicated to high titers in MEF cell lines independent of CatB and/or CatL (Fig. 3B,C). Therefore, we used the mouse disease model to investigate the effect of these cathepsins on ZEBOV replication *in vivo*. C57BL/6 mice (genetic background), CatB or CatL knockout mice (*catB^−/−^* or *catL^−/−^*) did not show an increased susceptibility to viral infection in general as determined here with VSV (Fig. 4C,D). In contrast, all knockout and C57BL/6 mice infected with MA-ZEBOV succumbed to infection with no difference in disease progression and time to death (Fig. 4A) or viral loads in liver, spleen and blood (Fig. 5) demonstrating that MA-ZEBOV replication *in vivo* is CatB- and CatL-independent.

In conclusion, our studies indicate that CatB and CatL are not absolutely required for EBOV replication. For yet unknown reasons, CatB seems to play a more considerable role in ZEBOV uptake than it does for any other EBOV species. EBOV seems to have evolved to use a broader spectrum of endosomal proteases to ensure GP cleavage and thus facilitates successful infection of target cells. Therefore, therapeutic approaches targeting single proteases are unlikely to be beneficial to combat EBOV infections.
